# Use of Generative AI for Improving Health Literacy in Reproductive Health: Case Study

**DOI:** 10.2196/59434

**Published:** 2024-08-06

**Authors:** Christina Burns, Angela Bakaj, Amonda Berishaj, Vagelis Hristidis, Pamela Deak, Ozlem Equils

**Affiliations:** 1 MiOra Encino, CA United States; 2 University of California San Diego San Diego, CA United States; 3 Institute for Management & Innovation University of Toronto Toronto, ON Canada; 4 College of Professional Studies Northeastern University Boston, MA United States; 5 Computer Science and Engineering University of California Riverside Riverside, CA United States; 6 Department of Obstetrics, Gynecology and Reproductive Sciences University of California San Diego San Diego, CA United States

**Keywords:** ChatGPT, chatGPT, chat-GPT, chatbots, chat-bot, chat-bots, artificial intelligence, AI, machine learning, ML, large language model, large language models, LLM, LLMs, natural language processing, NLP, deep learning, chatbot, Google Search, internet, communication, English proficiency, readability, health literacy, health information, health education, health related questions, health information seeking, health access, reproductive health, oral contraceptive, birth control, emergency contraceptive, comparison, clinical, patients

## Abstract

**Background:**

Patients find technology tools to be more approachable for seeking sensitive health-related information, such as reproductive health information. The inventive conversational ability of artificial intelligence (AI) chatbots, such as ChatGPT (OpenAI Inc), offers a potential means for patients to effectively locate answers to their health-related questions digitally.

**Objective:**

A pilot study was conducted to compare the novel ChatGPT with the existing Google Search technology for their ability to offer accurate, effective, and current information regarding proceeding action after missing a dose of oral contraceptive pill.

**Methods:**

A sequence of 11 questions, mimicking a patient inquiring about the action to take after missing a dose of an oral contraceptive pill, were input into ChatGPT as a cascade, given the conversational ability of ChatGPT. The questions were input into 4 different ChatGPT accounts, with the account holders being of various demographics, to evaluate potential differences and biases in the responses given to different account holders. The leading question, “what should I do if I missed a day of my oral contraception birth control?” alone was then input into Google Search, given its nonconversational nature. The results from the ChatGPT questions and the Google Search results for the leading question were evaluated on their readability, accuracy, and effective delivery of information.

**Results:**

The ChatGPT results were determined to be at an overall higher-grade reading level, with a longer reading duration, less accurate, less current, and with a less effective delivery of information. In contrast, the Google Search resulting answer box and snippets were at a lower-grade reading level, shorter reading duration, more current, able to reference the origin of the information (transparent), and provided the information in various formats in addition to text.

**Conclusions:**

ChatGPT has room for improvement in accuracy, transparency, recency, and reliability before it can equitably be implemented into health care information delivery and provide the potential benefits it poses. However, AI may be used as a tool for providers to educate their patients in preferred, creative, and efficient ways, such as using AI to generate accessible short educational videos from health care provider-vetted information. Larger studies representing a diverse group of users are needed.

## Introduction

Generative artificial intelligence (GenAI) is a widely used form of artificial intelligence (AI) that is particularly novel in nature given its ability to generate original outputs that have not been directly programmed by the algorithm’s designer [1]. Further, GenAI technology is capable of producing reports, videos, and music much faster and at a significantly lower cost, which may help to reduce societal inequities [2]. Porter and Zingaro have recently proposed that AI-assisted learning must be considered in the development of teaching methodologies [2].

In addition to GenAI, conversational AI platforms, such as OpenAI’s ChatGPT and Google’s Gemini, have been shown to have promising applications in health care. ChatGPT has been evaluated to provide informed, largely accurate, and well-articulated precursory responses to medical-related questions [3]. The conversational ability of AI has been suggested to provide empathetic and thorough responses to medical questions, sent by patients to their provider’s communication portal, which suggests its potential to be a technology that works to mitigate health care inequalities [4].

Recent studies have shown that individuals prefer using social media over their primary providers to locate reproductive health information [5]. The nonhuman nature of conversational AI technology may serve a pragmatic role in health care by providing a platform for patients to comfortably discuss sensitive topics such as sexual and reproductive health [6]. Recently, Burke-Garcia and Hicks [7] proposed that “Health Communication AI” may effectively counter chatbot health misinformation at a large scale and promote health. This is particularly seen in areas where there is a high level of controversy such as vaccines and where there is a need for large-scale individualized responses.

Here, we investigated the potential for ChatGPT (version 3.5) to be used as a first-line option for individuals to find reproductive health information and compared it to the existing Google Search technology. These free platforms were selected to test due to their accessibility to larger populations. We assessed the responses to reproductive health questions for themes of accuracy, effective communication delivery, and potential bias. Overall, Google responses were found to be better than ChatGPT responses in the evaluated categories. However, ChatGPT offered a more beneficial interface and ability to mimic a conversation, in comparison to inquiries being regarded as individual with Google Search. AI in general has the potential to be a useful tool for physicians to deliver information to their patients but was not deemed to be a better alternative for patients seeking answers to their health-related questions.

## Methods

### Study Design

We conducted a case study by asking a cascade of questions to ChatGPT (version 3.5). These questions were designed to be conversational and mimic a patient seeking information after they had missed a dose of oral contraceptive pill (OCP). The question sequence input into ChatGPT corresponded to the information flow found on the Planned Parenthood and Centers for Disease Control and Prevention websites regarding a missed dose of OCP [8,9].

In total, 11 questions were asked to ChatGPT in a question-answer format to mirror a patient-provider conversation, given the ability of AI to build off prior queries (Table S2 in Multimedia Appendix 1). Proceeding questions were developed from the ChatGPT response to the prior question. The questions were as follows: (1) What should I do if I missed a day of my oral contraception birth control? (2) What should I tell my health care provider? (3) Can you answer my concerns and questions instead of my doctor? (4) Where can I obtain emergency contraception? (5) Can you find a location near me to obtain emergency contraception? (6) Is emergency contraception covered by insurance? How much does it cost? (7) What do I do if I missed my period after forgetting to take a day of my oral contraception? (8) What happens if I resume taking my pills, but I really am pregnant? (9) Can you help me find a health care provider? (10) What kind of health care provider do I need to see for this issue? (11) Do you remember our conversation about oral contraception? (These questions were asked between 10 minutes and 1 week after the initial conversation of questions 1-10.)

In total, 2 researchers input these questions into ChatGPT over 1 week in February 2024. The questions were submitted through ChatGPT accounts owned by 4 different individuals, using their respective personal devices: an iPhone or MacBook laptop. The ChatGPT accounts were accessed via the ChatGPT iPhone app or the web-based ChatGPT version through the Safari (Apple Inc) internet browser. The 4 account holders had varying demographics: age, gender, educational background, primary language, profession, social media use, and internet use (Table 1). The same question cascade was entered into each ChatGPT account.

ChatGPT, which was released by OpenAI in 2022, has been trained using a reinforcement learning with human feedback model to generate responses that mimic human behavior [10]. In contrast to a computer program, which is limited to algorithms that produce an output to a given input, this AI program is capable of outputting results based on processing information in a way that mimics the human neural network [1]. With each additional use, it can improve and refine its outputs, separating it from a traditional computer and classifying it as intelligent [1]. In contrast, Google Search functions based on an algorithm that responds to queries with content it has gathered from the internet. This content is assigned a score based on a ranking algorithm, which yields the ranking of results generated from a given input [11,12].

**Figure 1:**
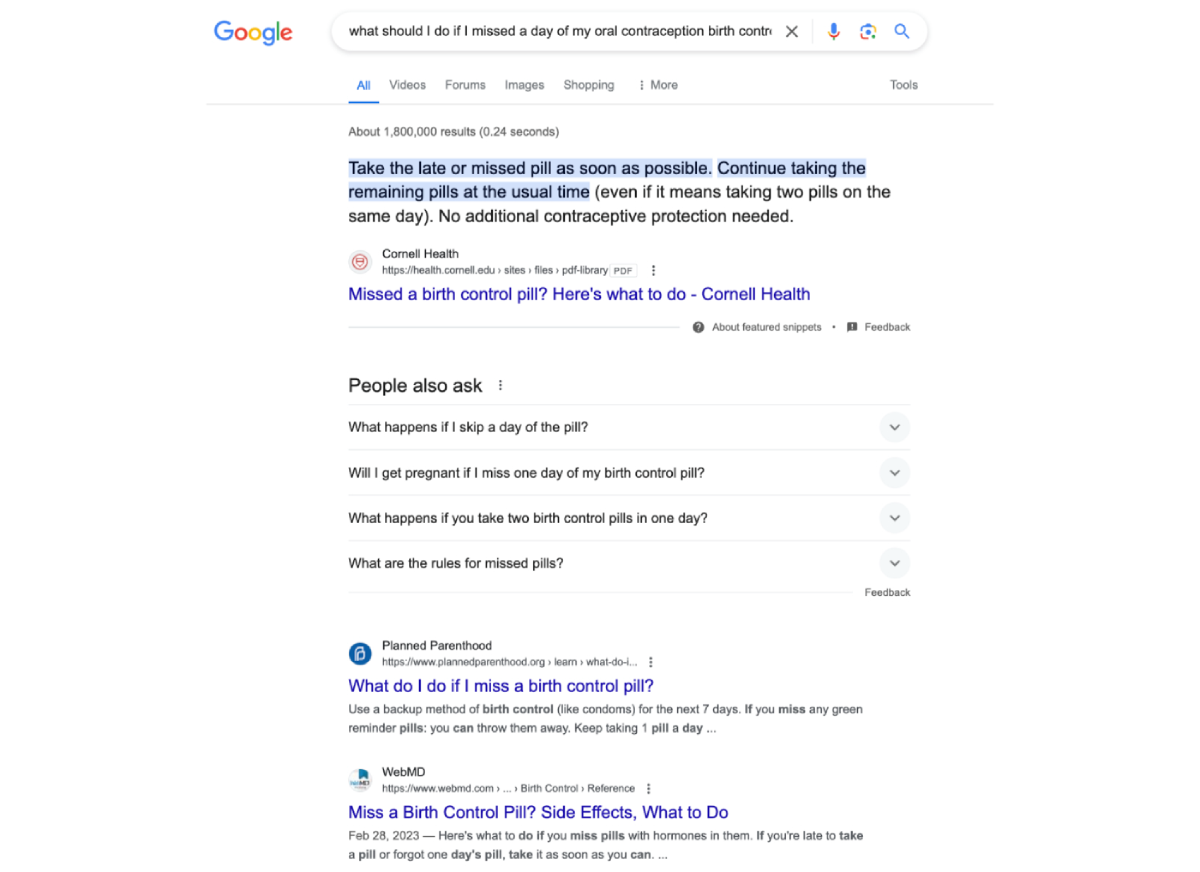
Vertical domains (All, Videos, Forums, etc), answer box (the top highlighted 3 lines), and snippet results (ie, the page links) for the Google Search inquiry.

Based on the variation between processing systems and given that Google Search is used by 77% of Americans to obtain health information [13], we compared the results from ChatGPT to Google Search results. This was performed by posing the first question, “what should I do if I missed a day of my oral contraception birth control?” to Google Search. This was repeated on 2 Safari browsers, 1 accessed using an iPhone and 1 accessed using a MacBook laptop. The first 7 Google Search results displayed at the top of the results page were compared to the ChatGPT responses. The first provided result is referred to as an answer box [11], with the following results referred to as snippets [14]. Further, the information on Google vertical domains (all, videos, forums, images, shopping, and more) were analyzed for content and format (Figure 1).

**Table 1:** The demographic information of the 4 ChatGPT account holders.

ChatGPT account	Age (years)	Gender identity	Educational background	Primary language English (yes or no)	Profession	Route to access ChatGPT	Social media use (daily average in minutes)	Internet use (daily average in minutes)
1	22	Female	Bachelor’s degree	Yes	Science	Safari internet browser	183	26
2	24	Female	Master’s degree	Yes	Science	ChatGPT iPhone app	77	18
3	76	Female	Elementary	No	Not science	ChatGPT iPhone app	21	0
4	55	Male	Bachelor’s degree	Yes	Not science	Safari internet browser	22	56

### Readability of the Responses

A health communication expert was consulted to determine the potential effectiveness of ChatGPT and Google Search responses to improve health literacy. To do this, the word count, the Flesch-Kincaid Grade Level [15], and the average reading time, in seconds, based on word count [16], were determined. These assessments were performed for each ChatGPT response to all 4 accounts, as well as for the preview text and full website link of the first 7 Google Search results. The effectiveness of information delivery was determined by assessing the vertical domains the information was delivered through, including written text, video, audio, and diagram formats [11].

### Medical Accuracy and Transparency of the Responses

A physician-researcher with a specialization in obstetrics and gynecology (OBGYN) assessed the ChatGPT and Google Search responses. They were evaluated for overall accuracy and similarity to information delivery standards outlined by the American College of Obstetrics and Gynecology guidelines regarding oral contraceptives. The OBGYN assessments were recorded in a descriptive format. The researchers also assessed whether the tools encouraged the user to seek expert medical care. ChatGPT and Google Search were also evaluated for transparency of data source and the reference publication date.

### Ethical Considerations

Per the HIPAA (Health Insurance Portability and Accountability Act), the participants’ identities were disclosed to the researchers only. This study’s data was deidentified and anonymously reported. This data collection is exempt under Western-Copernicus Group Institutional Review Board (1755408).

## Results

### Overview

The ChatGPT responses were more suitable for voice-enabled platforms, such as Alexa (Amazon.com) or Siri (Apple Inc), and for smaller mobile phone displays. The word count, Flesh-Kincaid score, and the reading time were determined for every ChatGPT response to each account. The Flesh-Kincaid score was 12.58 (SD 1.44) for account 1, 12.75 (SD 2.57) for account 2, 13.13 (SD 1.67) for account 3, and 13.93 (SD 2.28) for account 4 averaged across all the responses received by each account (Table S1 in Multimedia Appendix 1). Similarly, the reading time of all responses was averaged to be 56 (SD 18.35) seconds for account 1, 56.92 (SD 28.81) seconds for account 2, 17.17 (SD 4.39) seconds for account 3, and 48.42 (SD 21.72) seconds for account 4 (Table S1 in Multimedia Appendix 1).

ChatGPT responses to account holders with a science background were longer and more detailed. The ChatGPT response to the non–English-speaking account holder with a low level of education was the shortest. When asked about the default English proficiency for its responses, ChatGPT stated that it responds at a high school English proficiency, but that it can adjust the language complexity based on user preferences (Table S3 in Multimedia Appendix 1). ChatGPT provided the same response to all iPhone accounts regardless of the user’s age or gender.

All ChatGPT responses were in readable text format. When asked, ChatGPT informed the researchers that it cannot provide visual displays. There were no references or sources provided, even upon request. Only when asked, ChatGPT informed the researcher that the data were last updated in January 2022. At the end of each response, ChatGPT referred the researcher to a health care provider to obtain the most accurate information. A complete table of the ChatGPT responses to the tested questions can be found in Tables S2 and S3 in Multimedia Appendix 1.

The Google Search results listed links of broad types, including academic, government public health agency, and Planned Parenthood (Figures S1-S7 in Multimedia Appendix 1). Google Search delivered the answers in multiple formats by including figures, tables, and YouTube videos. Google Search results offered more comprehensive answers, given that they often combined a direct answer to the question, an answer box, and snippets for additional pages and references. The vertical domains included links to videos (YouTube videos on what to do if a day of oral contraceptive is missed), images (infographics and tables on oral contraceptives), shopping (where to purchase oral contraception), news (news articles from media sites such as CNN [Cable News Network] and BBC [British Broadcasting Corporation] addressing the question), and books (links to books written on oral contraception).

### Assessment of Accuracy

The OBGYN specialist compared the ChatGPT and Google Search responses to the current OBGYN standard of care and the American College of Obstetricians and Gynecologists informational materials [17]. Through this comparison, it was reported that ChatGPT responses were overall less accurate. Further, the ChatGPT responses were deemed to be poorly organized, vague, confusing, and included excessive information. The Google Search responses, in contrast, were assessed to be clearer and more accurate.

### Assessment of Equitable Use

To evaluate whether ChatGPT and Google Search provided equitable benefits to individuals with varying levels of health literacy, the responses were evaluated using the recommendations of a health communication expert. The average word count of the ChatGPT responses was significantly longer than that of the Google Search answer box and snippets. The average Flesh-Kincaid score for the ChatGPT responses was 13.10 (SD 2.04), which corresponds to college-level reading ability, while the average score for the Google Search responses was 5.93 (SD 1.56), correlating with a middle school–level reading ability [15]. The average reading time of ChatGPT responses was longer than the average reading time for Google Search answer box and snippet responses (Table 2).

**Table 2:** Readability of ChatGPT versus Google Search responses to the question “what should I do if I missed a day of my oral contraception birth control?”

	Average word count, mean (SD)	Average Flesh-Kincaid Score (grade), mean (SD)	Average duration to read response (seconds), mean (SD)
ChatGPT	176.85 (101.83)	13.10 (2.04)	44.63 (18.32)
Google Search	34.14 (12.27)	5.93 (1.56)	8.86 (2.79)

## Discussion

In recent years, social media has become a broadly used source for health-related information by the community [18]. Health care organizations, providers, and the research industry have adopted the use of social media to deliver health knowledge and information [18]. Other technological tools such as search engines, such as Google and Bing (Microsoft), are being used commonly by laypeople seeking access to health information [19]. Additionally, health blogs are a popular technological tool among patients seeking answers to their health-related questions [4]. Further, AI has been increasingly used in health care to improve patient care [20]. Health consortiums such as Kaiser Permanente have implemented AI into their health care delivery to aid in identifying high-risk patients [21]. AI has also been used to reduce physician burnout and improve physician-patient interactions by filtering through providers’ overcrowded message inboxes [22].

We observed that, in the current format, ChatGPT (version 3.5) responses were overall less accurate, less up-to-date, less transparent, and less user-friendly compared to the Google Search results. Google Search provided clearer, more current, and accurate responses, while including references and delivering through multiple user-friendly platforms, or vertical domains. However, these responses were not individualized to the user, given that Google Search does not view subsequent queries as part of the same conversation. This limits Google Search from maintaining context as well as ChatGPT in mimicking a patient asking follow-up questions to a provider [23]. Another concern with Google Search is that although clearly labeled, sponsored and advertised messages may be high on the results display [24].

ChatGPT appeared to have access to some user information, as determined by the varying length and depth of ChatGPT responses to different device accounts, depending on demographics. However, Google Search provided the same response content across devices. There was no information provided on the extent of user information ChatGPT accesses. A challenge in using public tools such as ChatGPT, Microsoft Copilot, or Gemini, is that they do not offer any privacy guarantees, nor are they HIPAA compliant. It is possible to build GenAI tools that are private; however, they require a significant amount of funding and time [25], and a paid platform may not be accessible to low-income users.

Transparency is critical to gain public trust [26]. Improved transparency by providing references for the responses and obtaining approval for user preferences is important for the implementation of ChatGPT. This is particularly important for groups with a preexisting elevated level of distrust. ChatGPT is trained on specific data sets, which are not clearly disclosed and may not have adequate diversity and representation. Consequently, this could present biases and gaps in ChatGPT performance for marginalized populations. Additionally, GenAI platforms can create incorrect facts called “hallucinations” [27], which may lead to higher levels of public distrust. ChatGPT also has the potential to lead to large-scale medical errors with legal implications [28].

Currently, the intended role of ChatGPT in health care is unclear. Section 201(h) of the Food, Drug, and Cosmetic Act issued by the Food and Drug Administration (FDA) defines a medical device as a tool intended for use in the diagnosis of disease or other conditions, or in the cure, mitigation, treatment, or prevention of disease in man or other animals and does not achieve its primary purposes through chemical action within or on the body [29]. If ChatGPT is to be used as a medical device, it must be held to the standards of FDA regulations. The FDA requires medical devices to meet specific accuracy thresholds and disclose them as such. Currently, there is no clear data on the accuracy of ChatGPT tools. The FDA also requires medical device manufacturers to provide phone numbers for “the medical equipment supplier, the home health care agency, the doctor, the referral for disposal of the device, and/or any other appropriate points of contact for the typical user” [30]. At the end of each response, ChatGPT suggested that the user confirm the recommendation with a health care provider (Table S1 in Multimedia Appendix 1). However, this statement does not eliminate ChatGPT’s responsibility to the user, and leaves room for ChatGPT to meet the outlined FDA requirements by providing direct health care contact information to the user.

To address these concerns, Burke-Garcia and Hicks [7] proposed that public health experts, health communicators, and providers collaborate with technology innovators, develop new or adapt existing health communication theories, and test and evaluate these tools to ensure they can adapt to unique cultural, historical, linguistic, and regional contexts, or develop incorporating domain expertise. One way to overcome these challenges is the use of AI by health care providers rather than patients. We tested whether a provider could combine Google Search and AI to generate individualized, accurate, and transparent health information material that may be used to educate a patient. Currently, health care providers are limited to static information sheets and pamphlets that are not specific to the individual patient. These written informational materials may also exclude individuals with low health literacy and English proficiency. According to a Gallup survey in 2020, 54% of adults in the United States had English literacy below 6th grade [31]. Similarly, the US Department of Education reports that approximately 1 in 5 US adults has a Program for the International Assessment of Adult Competencies literacy scale (measures literacy, numeracy, and digital problem-solving skills) below level 1, indicating low English literacy [32]. In addition, a previous study shows that patients prefer watching a video over reading a pamphlet and that educational videos boost shared decision-making in clinical practice while increasing health literacy and medication adherence [33].

To address these considerations and to test whether AI technology may be used in a clinic setting to generate educational materials, the first Google Search result in response to the leading OCP question was developed into an AI-generated short video. The video was created in both English and Spanish, with closed captions, using the free version of an AI tool (Multimedia Appendices 2 and 3) Invideo. The app generated a short video efficiently, in less than 3 minutes. The English video was found to accurately convey the input text and was easy to comprehend. The Spanish video was deemed to be identical in message by 2 native Spanish-speaking researchers. These data suggest that AI-generated videos can be easily and efficiently produced by providers in a clinic setting to be shared with the patient and their families. This may help reduce physician burnout [22], improve provider-patient communication, and increase health literacy, especially in situations with a language barrier between the patient and provider.

This pilot study has several limitations. We used ChatGPT (version 3.5) which has fewer features compared to OpenAI’s more recent and paid version, ChatGPT (version 4). ChatGPT (version 4) can analyze and comment on uploaded images and audio, has improved accuracy, has a longer context window, and processes data faster than ChatGPT (version 3.5). However, the paid version of ChatGPT may not be accessible to people with lower socioeconomic status, which may lead to discrepancies in the information provided to different populations.

Another limitation of this study is that ChatGPT was tested using only 4 different accounts. To overcome this limitation, we used accounts of 4 diverse individuals: young versus old, male versus female, child-bearing age versus postmenopausal, American-born versus immigrant, English-speaking versus non–English-speaking, and college-educated versus primary school educated. Additionally, in our study, the clarity of ChatGPT and Google Search responses was evaluated by experts in health care and science. Current research on the quality of health care apps of ChatGPT relies on experts’ assessments rather than patients’ assessments [34], which may lead to inaccurate conclusions. Our study attempted to overcome this bias by using validated measures such as word count, the Flesch-Kincaid Grade Level assessment, and the average reading time, in seconds, based on word count. Future studies need to assess the user experience among a larger cohort of people from diverse demographics, and educational, regional, and socioeconomic backgrounds.

Our study identified Google Search to be a more thorough, accurate, current, transparent, and user-friendly health informatics tool, compared to ChatGPT. We also observed that providers may use video or audio-generating AI tools to convert provider-vetted Google Search results into personalized and patient-specific health infographics. These infographics may be shared with the patient during the clinic visit to improve patient-provider communication and patient health literacy, particularly in settings with language barriers present. This approach would secure an ethical, equitable, and accurate use of AI to improve patient health knowledge.

The rapid and ongoing evolution of AI tools for health care, combined with the lack of clear regulatory frameworks to assess the safety and effectiveness of different versions underscores the urgent need for collaboration between FDA, health care professionals, and technology developers [35]. Burke-Garcia and Hicks [7] propose that a strong partnership between medical and health communication specialists and technology innovators is essential to developing a health communication AI that can adapt to diverse cultural, educational, linguistic, and historical contexts. Furthermore, new or adapted health communication theories and frameworks must be piloted and tested. It is also important to train health experts in the theory and application of AI [35]. All these efforts require funding, which could be incorporated into the AI development costs [7].

## Appendix

Multimedia Appendix 1 Full data set appendix.

Multimedia Appendix 2 AI-generated video in English using the Google Search response to the question “what should I do if I missed a day of my oral contraception birth control?”

Multimedia Appendix 3 AI-generated video in Spanish using the Google Search response to the question “what should I do if I missed a day of my oral contraception birth control?”

## Abbreviations

AI: artificial intelligence
BBC: British Broadcasting Corporation
CNN: Cable News Network
FDA: Food and Drug Administration
GenAI: generative artificial intelligence
HIPAA: Health Insurance Portability and Accountability Act
OBGYN: obstetrics and gynecology
OCP: oral contraceptive pill

## Data Availability

The full data set of responses is publicly available in Multimedia Appendix 1.

## References

[1] Xu Y, Liu X, Cao X, Huang C, Liu E, Qian S, Liu X, Wu Y, Dong F, Qiu C, Qiu J, Hua K, Su W, Wu J, Xu H, Han Y, Fu C, Yin Z, Liu M, Roepman R, Dietmann S, Virta M, Kengara F, Zhang Z, Zhang L, Zhao T, Dai J, Yang J, Lan L, Luo M, Liu Z, An T, Zhang B, He X, Cong S, Liu X, Zhang W, Lewis JP, Tiedje JM, Wang Q, An Z, Wang F, Zhang L, Huang T, Lu C, Cai Z, Wang F, Zhang J (2021). Artificial intelligence: a powerful paradigm for scientific research. Innovation (Camb).

[2] Ismael K, Patringenaru I, Clementi K (2023). In this era of AI, will everyone be a programmer? [Internet].

[3] Grünebaum A, Chervenak J, Pollet SL, Katz A, Chervenak FA (2023). The exciting potential for ChatGPT in obstetrics and gynecology. Am J Obstet Gynecol.

[4] Ayers JW, Poliak A, Dredze M, Leas EC, Zhu Z, Kelley JB, Faix DJ, Goodman AM, Longhurst CA, Hogarth M, Smith DM (2023). Comparing physician and artificial intelligence chatbot responses to patient questions posted to a public social media forum. JAMA Intern Med.

[5] Gabarron E, Wynn R (2016). Use of social media for sexual health promotion: a scoping review. Glob Health Action.

[6] Ryan KL, Arbuckle-Bernstein V, Smith G, Phillips J (2018). Let's talk about sex: a survey of patients' preferences when addressing sexual health concerns in a family medicine residency program office. PRiMER.

[7] Burke-Garcia A, Hicks RS (2024). Scaling the idea of opinion leadership to address health misinformation: the case for "Health Communication AI". J Health Commun.

[8] What do I do if I miss a birth control pill? [Internet]. Planned Parenthood.

[9] Recommended actions after late or missed combined oral contraceptives.

[10] What is ChatGPT? [Internet]. OpenAI.

[11] Hristidis V, Ruggiano N, Brown EL, Ganta SRR, Stewart S (2023). ChatGPT vs Google for queries related to dementia and other cognitive decline: comparison of results. J Med Internet Res.

[12] Levene M (2010). Search engines: information retrieval in practice. Comput J.

[13] Cai HC, King LE, Dwyer JT (2021). Using the Google™ search engine for health information: is there a problem? Case study: supplements for cancer. Curr Dev Nutr.

[14] (2024). Featured snippets and your website [Internet]. Google Search Central.

[15] Flesch reading ease and the flesch kincaid grade level [Internet]. Readable.

[16] Words to time converter [Internet]. The Read Time.

[17] Birth Control. American College of Obstetricians and Gynecologists.

[18] Chen J, Wang Y (2021). Social media use for health purposes: systematic review. J Med Internet Res.

[19] Wang L, Wang J, Wang M, Li Y, Liang Y, Xu D (2012). Using internet search engines to obtain medical information: a comparative study. J Med Internet Res.

[20] Mintz Y, Brodie R (2019). Introduction to artificial intelligence in medicine. Minim Invasive Ther Allied Technol.

[21] Martinez VA, Betts RK, Scruth EA, Buckley JD, Cadiz VR, Bertrand LD, Paulson SS, Dummett BA, Abhyankar SS, Reyes VM, Hatton JR, Sulit R, Liu VX (2022). The Kaiser Permanente Northern California Advance Alert Monitor Program: an automated early warning system for adults at risk for in-hospital clinical deterioration. Jt Comm J Qual Patient Saf.

[22] Tierney AA, Gayre G, Hoberman B, Mattern B, Ballesca M, Kipnis P, Liu V, Lee K (2024). Ambient artificial intelligence scribes to alleviate the burden of clinical documentation. NEJM Catalyst.

[23] OpenAI Memory and new controls for ChatGPT [Internet]. OpenAI.

[24] Get your ads to show on the first page of Google search results [Internet]. Google.

[25] OpenAI Introducing ChatGPT enterprise [Internet]. 2023.

[26] Séroussi B, Hollis KF, Soualmia LF (2020). Transparency of health informatics processes as the condition of healthcare professionals' and patients' trust and adoption: the rise of ethical requirements. Yearb Med Inform.

[27] Alkaissi H, McFarlane SI (2023). Artificial hallucinations in ChatGPT: implications in scientific writing. Cureus.

[28] Athaluri SA, Manthena SV, Kesapragada VSRKM, Yarlagadda V, Dave T, Duddumpudi RTS (2023). Exploring the boundaries of reality: investigating the phenomenon of artificial intelligence hallucination in scientific writing through ChatGPT references. Cureus.

[29] Office of the Commissioner, Office of Special Medical Programs, Office of Combination Products, United States Department of Health and Human Services, United States Food and Drug Administration, Office of the Commissioner, Office of Special Medical Programs, Office of Combination Products (2017). Classification of products as drugs and devices and additional product classification issues: guidance for industry and FDA staff.

[30] Labeling Research and Policy Development Branch, Division of Device User Programs and Systems Analysis, Office of Health and Industry Programs, U.S. Department of Health and Human Services, Food and Drug Administration, Center for Devices and Radiological Health, Labeling Research and Policy Development Branch, Division of Device User Programs and Systems Analysis (2006). Guidance on medical device patient labeling; final guidance for industry and FDA reviewers.

[31] Rothwell J Assessing the economic gains of eradicating illiteracy nationally and regionally in the United States [Internet]. 2020.

[32] National Center for Education Statistics (2019). Adult literacy in the United States [Internet]. U.S. Department of Education NCES.

[33] Hung C, Chen Y, Hung T, Chiang C, Chen C, Wang WM (2022). Clinician-created educational video for shared decision-making in the outpatient management of acne. PLoS One.

[34] Hackl E (2024). Assessing ChatGPT's use of person-first language in healthcare conversations. Discov Artif Intell.

[35] How FDA regulates artificial intelligence in medical products. The Pew Charitable Trusts.

