# Supplementary material for: Use of Generative AI for Improving Health Literacy in Reproductive Health: Case Study
Source: JMIR Form Res. 2024 Aug 6;8:e59434. doi: 10.2196/59434 (PMC11336497; doi:10.2196/59434)
Supplement: Multimedia Appendix 1 [file formative_v8i1e59434_app1.docx]

**Table S1.** Comparison of ChatGPT responses as a function of account.

|  | **Account** | | | |
| --- | --- | --- | --- | --- |
| **Question** | **1** | **2** | **3** | **4** |
| What should I do if I missed a day of my oral contraception birth control? | Word Count: 246 | Word Count: 277 | Word Count: 81 | Word Count: 212 |
|  | Flesh Kincaid Reading Level: 11.6 | Flesh Kincaid Reading Level: 10.4 | Flesh Kincaid Reading Level: 10.4 | Flesh Kincaid Reading Level: 12.5 |
|  | Reading Time (s): 62 | Reading Time (s): 70 | Reading Time (s): 20 | Reading Time (s): 53 |
| What should I tell my healthcare provider? | Word Count: 257 | Word Count: 272 | Word Count: 76 | Word Count: 191 |
|  | Flesh Kincaid Reading Level: 13.5 | Flesh Kincaid Reading Level: 13.7 | Flesh Kincaid Reading Level: 11.8 | Flesh Kincaid Reading Level: 14.4 |
|  | Reading Time (s): 65 | Reading Time (s): 69 | Reading Time (s): 19 | Reading Time (s): 48 |
| Can you answer my concerns and questions instead of my doctor? | Word Count: 274 | Word Count: 49 | Word Count: 53 | Word Count: 77 |
|  | Flesh Kincaid Reading Level: 10.8 | Flesh Kincaid Reading Level: 12.0 | Flesh Kincaid Reading Level: 13.3 | Flesh Kincaid Reading Level: 19.2 |
|  | Reading Time (s): 69 | Reading Time (s): 12 | Reading Time (s): 13 | Reading Time (s): 23 |
| Where can I get emergency contraception? | Word Count: 224 | Word Count: 259 | Word Count: 80 | Word Count: 197 |
|  | Flesh Kincaid Reading Level: 14.3 | Flesh Kincaid Reading Level: 12.9 | Flesh Kincaid Reading Level: 13.3 | Flesh Kincaid Reading Level: 12.8 |
|  | Reading Time (s): 56 | Reading Time (s): 65 | Reading Time (s): 20 | Reading Time (s): 50 |
| Can you find a location near me to get emergency contraception? | Word Count: 199 | Word Count: 259 | Word Count: 60 | Word Count: 173 |
|  | Flesh Kincaid Reading Level: 11.5 | Flesh Kincaid Reading Level: 14.0 | Flesh Kincaid Reading Level: 14.2 | Flesh Kincaid Reading Level: 13.6 |
|  | Reading Time (s): 50 | Reading Time (s): 65 | Reading Time (s): 15 | Reading Time (s): 44 |
| Is emergency contraception covered by insurance? How much does it cost? | Word Count: 262 | Word Count: 233 | Word Count: 107 | Word Count: 218 |
|  | Flesh Kincaid Reading Level: 14.0 | Flesh Kincaid Reading Level: 13.3 | Flesh Kincaid Reading Level: 11.6 | Flesh Kincaid Reading Level: 14.1 |
|  | Reading Time (s): 66 | Reading Time (s): 56 | Reading Time (s): 27 | Reading Time (s): 55 |
| What do I do if I missed my period after forgetting to take a day of my oral contraception? | Word Count: 259 | Word Count: 290 | Word Count: 74 | Word Count: 279 |
|  | Flesh Kincaid Reading Level: 10.1 | Flesh Kincaid Reading Level: 14.4 | Flesh Kincaid Reading Level: 13.3 | Flesh Kincaid Reading Level: 15.7 |
|  | Reading Time (s): 65 | Reading Time (s): 73 | Reading Time (s): 19 | Reading Time (s): 70 |
| What happens if I resume my pills, but I really am pregnant? | Word Count: 268 | Word Count: 331 | Word Count: 68 | Word Count: 245 |
|  | Flesh Kincaid Reading Level: 12.7 | Flesh Kincaid Reading Level: 16.1 | Flesh Kincaid Reading Level: 12.9 | Flesh Kincaid Reading Level: 13.8 |
|  | Reading Time (s): 68 | Reading Time (s): 83 | Reading Time (s): 17 | Reading Time (s): 62 |
| Can you help me find a healthcare provider? | Word Count: 260 | Word Count: 365 | Word Count: 61 | Word Count: 262 |
|  | Flesh Kincaid Reading Level: 11.8 | Flesh Kincaid Reading Level: 12.1 | Flesh Kincaid Reading Level: 14.7 | Flesh Kincaid Reading Level: 13.6 |
|  | Reading Time (s): 66 | Reading Time (s): 92 | Reading Time (s): 15 | Reading Time (s): 66 |
| What kind of healthcare provider do I need to see for this issue? | Word Count: 265 | Word Count: 296 | Word Count: 70 | Word Count: 327 |
|  | Flesh Kincaid Reading Level: 14.6 | Flesh Kincaid Reading Level: 16.6 | Flesh Kincaid Reading Level: 15.8 | Flesh Kincaid Reading Level: 16.0 |
|  | Reading Time (s): 67 | Reading Time (s): 75 | Reading Time (s): 18 | Reading Time (s): 82 |
| Ten minutes to one week later: Do you remember our conversation about oral contraception? | Word Count: 47 | Word Count: 29 | Word Count: 47 | Word Count: 55 |
|  | Flesh Kincaid Reading Level: 13.4 | Flesh Kincaid Reading Level: 7.6 | Flesh Kincaid Reading Level: 10.9 | Flesh Kincaid Reading Level: 10.7 |
|  | Reading Time (s): 12 | Reading Time (s): 7 | Reading Time (s): 12 | Reading Time (s): 14 |
| Does your memory about our past conversations influence the answers you give to new questions? | Word Count: 105 | Word Count: 62 | Word Count: 44 | Word Count: 54 |
|  | Flesh Kincaid Reading Level: 12.7 | Flesh Kincaid Reading Level: 9.9 | Flesh Kincaid Reading Level: 14.8 | Flesh Kincaid Reading Level: 11.1 |
|  | Reading Time (s): 26 | Reading Time (s): 16 | Reading Time (s): 11 | Reading Time (s): 14 |

**Table S2.** ChatGPT missed oral contraceptive Inquiry.

|  | Account | | | |
| --- | --- | --- | --- | --- |
| Question | 1 | 2 | 3 | 4 |
| What should I do if I missed a day of my oral contraception birth control? | If you miss a day of your oral contraception (birth control pill), it's important to follow the guidelines provided by your healthcare provider or the instructions in the package insert. Generally, the steps to take if you miss a pill include:  **Take the Missed Pill ASAP:** If you realize you missed a pill, take it as soon as you remember. If it's close to the time for your next scheduled dose, take both the missed pill and the regular pill at that time.  **Use Backup Contraception:** Consider using a backup method of contraception (such as condoms) for the next 7 days if you have unprotected sex during this time.  **Follow Specific Instructions:** The exact steps can vary depending on the type of birth control pill you're taking, so it's crucial to refer to the specific instructions provided by your healthcare provider or the pill packaging.  **Emergency Contraception:** If you had unprotected sex in the last 72 hours and missed multiple pills, consider using emergency contraception. Consult your healthcare provider for advice.  **Contact Your Healthcare Provider:** If you're unsure about what to do or if you have any concerns, it's important to contact your healthcare provider. They can provide personalized guidance based on your specific situation.  Remember, consistency is crucial for the effectiveness of oral contraceptives. If you continue to miss pills or have concerns about your birth control method, talk to your healthcare provider about alternative options or strategies that may be more suitable for you. | If you've missed a dose of your oral contraception (birth control pill), it's important to take action to minimize the risk of pregnancy. Here's what you should do:    1. **Take the Missed Pill ASAP**: Take the missed pill as soon as you remember, even if it means taking two pills in one day. If you've missed more than one pill, only take one extra pill.    2. **Follow Regular Schedule**: Continue taking the rest of the pills at your usual time, even if it means taking two pills in one day. This might mean taking two pills on the same day or one pill on two consecutive days.    3. **Use Backup Contraception**: If you've missed more than one pill or if you've missed a pill in the first week of your pill pack, it's recommended to use backup contraception, such as condoms, for the next seven days.    4. **Emergency Contraception**: If you've had unprotected sex during the time you missed your pills or within the last five days, you might consider emergency contraception. Emergency contraception can reduce the risk of pregnancy if taken within a certain timeframe after unprotected sex.    5. **Contact Your Healthcare Provider**: If you're unsure what to do or if you've missed multiple pills, it's best to contact your healthcare provider or pharmacist for guidance. They can provide personalized advice based on your specific situation.    Remember, missing a single pill doesn't necessarily mean you'll become pregnant, but it does increase the risk. Taking action promptly can help minimize that risk. Additionally, it's essential to use a backup method of contraception until you've taken your pills consistently for several days to ensure continued protection against pregnancy. | If you miss a day of your oral contraception (birth control pill), take the missed pill as soon as you remember, even if it means taking two pills in one day. Use a backup form of contraception, such as condoms, for the next seven days. Check the specific instructions for your brand of birth control pills or consult with your healthcare provider for personalized guidance. If you have concerns or experience any unusual symptoms, it's important to contact your healthcare professional. | If you miss a day of your oral contraception birth control, here's what you should generally do:  Take the Missed Pill ASAP: Take the missed pill as soon as you remember, even if it means taking two pills in one day. If you miss more than one pill, only take one extra pill.  Use Backup Contraception: Use backup contraception, such as condoms, for the next seven days after you've missed a pill, especially if you've had unprotected sex in the past five days.  Follow Package Instructions: Follow the instructions on your pill package or consult your healthcare provider for specific guidance based on the type of pill you're taking.  Consider Emergency Contraception: If you've had unprotected sex in the past five days and missed two or more pills, consider emergency contraception.  Continue Taking Pills as Usual: Continue taking the rest of your pills at your regular time, even if you're taking two pills in one day.  Seek Medical Advice if Needed: If you're unsure about what to do or have any concerns, it's best to consult your healthcare provider for personalized advice.  Remember, the effectiveness of oral contraception can be reduced if pills are missed or taken inconsistently, so it's important to follow the instructions carefully and use backup contraception if necessary. |
| What should I tell my healthcare provider | When you contact your healthcare provider after missing a day of your oral contraception, it's important to provide them with detailed information about your situation. Here are some key points to mention:  **Date and Time of Missed Pill:** Specify the date and time you missed the pill.  **Type of Pill:** Identify the specific brand and type of oral contraceptive you are taking, as different pills may have different instructions for missed doses.  **Actions Taken:** Describe the actions you've taken since realizing you missed the pill, such as whether you took the missed pill as soon as you remembered, and if you used backup contraception.  **Sexual Activity:** If you've had unprotected sex since missing the pill, provide details about any recent sexual activity, as this information will help your healthcare provider assess the risk of pregnancy.  **Any Other Medications:** Inform your healthcare provider about any other medications, supplements, or substances you are currently taking, as they may interact with your birth control.  **Concerns or Questions:** If you have specific concerns or questions related to your birth control, express them to your healthcare provider. This may include concerns about potential pregnancy, side effects, or the need for an alternative contraceptive method.  Remember, your healthcare provider is there to help and provide guidance. Open communication will allow them to assess your situation accurately and offer appropriate advice or adjustments to your contraceptive plan. If you are uncomfortable discussing these matters directly, consider writing down your concerns and questions beforehand to ensure that you cover all relevant information during your conversation. | When discussing the missed dose of your oral contraception with your healthcare provider, it's important to provide them with all relevant information so they can offer you the best guidance. Here's what you should tell them:    1. **Details of the Missed Dose**: Explain when you missed the dose(s) and how many pills you missed. Include any relevant information about whether you took the missed pill(s) as soon as you remembered and if you took additional pills to catch up.    2. **Current Symptoms or Concerns**: If you're experiencing any unusual symptoms or side effects, such as breakthrough bleeding, nausea, or breast tenderness, let your healthcare provider know. They can assess whether these symptoms are related to the missed dose or if further evaluation is needed.    3. **Recent Sexual Activity**: If you've had unprotected sex recently, provide details about when it occurred and whether you used any backup contraception. This information can help your healthcare provider assess your risk of pregnancy and whether emergency contraception might be necessary.    4. **Questions or Concerns**: If you have any questions or concerns about your birth control method, this is the time to discuss them with your healthcare provider. They can provide clarification, address any misconceptions, and offer guidance on using your contraception effectively.    5. **Other Medications or Supplements**: Inform your healthcare provider about any other medications, supplements, or herbal remedies you're currently taking, as these could potentially interact with your birth control pills.    By providing your healthcare provider with this information, they can offer you personalized advice and recommendations based on your specific situation and help ensure that you're using your birth control effectively to prevent pregnancy. | When discussing a missed dose of your oral contraception with your healthcare provider, be sure to mention the specific details, including the date and time you missed the pill. Describe any steps you've taken to remedy the situation, such as taking the missed pill as soon as you remembered. Additionally, mention if you used a backup form of contraception. This information will help your healthcare provider provide tailored advice and guidance based on your individual circumstances. | When discussing a missed dose of your oral contraception with your healthcare provider, it's important to provide them with the following information:  Details of the Missed Dose: Explain when you missed the dose, how many pills you missed, and whether you took the missed pill as soon as you remembered.  Any Additional Medications or Supplements: Inform your healthcare provider of any other medications or supplements you're currently taking, as they may interact with your birth control pills.  Recent Sexual Activity: If applicable, discuss any recent sexual activity you've had since missing the dose and whether you used backup contraception.  Concerns or Side Effects: Mention any concerns or side effects you may have experienced since missing the dose.  Future Prevention Strategies: Ask your healthcare provider for guidance on preventing pregnancy moving forward, including whether you need to use backup contraception and for how long.  Any Other Relevant Medical History: Provide any relevant medical history or conditions that may impact your contraception needs.  By providing your healthcare provider with this information, they can offer personalized advice and guidance on how to proceed to ensure effective contraception and address any concerns you may have. |
| Can you answer my concerns and questions instead of my doctor? | Certainly, I can provide general information, but it's important to note that I am not a substitute for professional medical advice. Your healthcare provider has access to your complete medical history and can provide personalized guidance based on your individual circumstances. That being said, I can offer some general information that may address common concerns related to missing a day of oral contraception:  **Risk of Pregnancy:**  The risk of pregnancy increases when you miss a pill, especially if it's not promptly addressed.  Taking the missed pill as soon as possible and using backup contraception for the next 7 days can help minimize the risk.  **Backup Contraception:**  Using condoms or another backup method is essential after missing a pill to prevent unintended pregnancy.  Ensure consistent and correct use of the backup method during the specified period.  **Emergency Contraception:**  If you had unprotected sex in the last 72 hours and missed multiple pills, emergency contraception may be considered. Consult with your healthcare provider promptly.  **Side Effects or Discomfort:**  Missing a pill might lead to hormonal fluctuations and could cause side effects like spotting or breakthrough bleeding.  If you experience any unusual symptoms or discomfort, discuss them with your healthcare provider.  **Adjustments to Birth Control Plan:**  Depending on your specific situation and the type of birth control pill you're taking, your healthcare provider may recommend adjustments to your contraceptive plan.  Remember, it's crucial to contact your healthcare provider directly to discuss your specific situation. They can offer personalized advice based on your health history and the details of your missed pill. If you're uncertain about anything, don't hesitate to reach out to your healthcare provider for guidance. | Of course, I'm here to help address your concerns and questions to the best of my ability. Please feel free to share any specific worries or uncertainties you have regarding your missed dose of oral contraception, and I'll do my best to provide you with relevant information and guidance. | While I can provide general information and answer questions, I am not a substitute for professional medical advice, diagnosis, or treatment. It's essential to consult with your healthcare provider for personalized guidance based on your specific health situation. If you have medical concerns, your doctor is the best resource to address them comprehensively. | While I can provide general information and guidance on various topics, including healthcare, it's important to remember that I am not a substitute for professional medical advice from a qualified healthcare provider. If you have specific concerns or questions about your health, especially regarding medications like birth control pills, it's always best to consult with a healthcare professional who can provide personalized advice based on your individual circumstances, medical history, and needs. They can offer tailored recommendations and address any concerns you may have in a comprehensive and medically appropriate manner. |
| Where can I get emergency contraception? | Emergency contraception, also known as the morning-after pill, is available without a prescription at various locations. Here are common places where you can obtain emergency contraception:  **Pharmacies:**  Most pharmacies, including chain and independent pharmacies, carry emergency contraception. It is typically available over the counter without the need for a prescription.  **Family Planning Clinics:**  Family planning clinics, reproductive health clinics, and sexual health clinics often provide emergency contraception. These clinics may also offer additional services and information about contraception.  **Healthcare Providers:**  You can obtain emergency contraception from your healthcare provider or gynecologist. They can also provide guidance on its use and answer any questions you may have.  **College Health Centers:**  If you are a student, many college and university health centers offer reproductive health services, including emergency contraception.  **Women's Health Organizations:**  Some women's health organizations or community health centers may provide emergency contraception services.  **Online Pharmacies:**  In some regions, you may be able to order emergency contraception online through reputable pharmacies. However, it's crucial to ensure the legitimacy and reliability of the source.  When obtaining emergency contraception, it's important to act quickly. The effectiveness of emergency contraception decreases over time, so it's recommended to take it as soon as possible after unprotected sex or contraceptive failure. If you have concerns or questions about emergency contraception, consider contacting a healthcare professional or a pharmacist for guidance. | Emergency contraception is available from several sources, including:    1. **Pharmacies**: Many pharmacies sell emergency contraception over the counter without a prescription. You can simply walk into a pharmacy and ask for emergency contraception. It's typically available in the family planning or women's health section.    2. **Health Clinics**: Health clinics, including Planned Parenthood clinics, often provide emergency contraception. You may need to schedule an appointment, but many clinics also offer walk-in services. These clinics may offer emergency contraception at a reduced cost or for free based on your income level.    3. **Healthcare Providers**: Your healthcare provider, such as your doctor or gynecologist, can prescribe emergency contraception for you. If you have a regular healthcare provider, you can contact their office to schedule an appointment. They may also provide guidance over the phone or via telehealth appointments.    4. **Online Pharmacies**: Some online pharmacies offer emergency contraception for purchase. However, it's important to ensure that you're using a reputable and licensed online pharmacy to purchase any medication.    5. **Emergency Rooms**: In some cases, emergency rooms may provide emergency contraception. However, this option is typically more expensive and should be reserved for situations where other options are not accessible.    It's important to note that emergency contraception should be used as soon as possible after unprotected sex or contraceptive failure for maximum effectiveness. The sooner you take it, the more likely it is to prevent pregnancy. If you're unsure where to access emergency contraception or have any questions about its use, don't hesitate to reach out to a healthcare provider or pharmacist for assistance. | You can obtain emergency contraception, often known as the "morning-after pill," from various sources. These include pharmacies, family planning clinics, and healthcare providers. In many places, it is available over-the-counter without a prescription. If you need emergency contraception, it's recommended to obtain it as soon as possible, preferably within 72 hours after unprotected intercourse. If you have specific questions about where to obtain emergency contraception in your location, consider reaching out to a local pharmacy or healthcare provider for guidance. | You can obtain emergency contraception, also known as the morning-after pill, from several places, including:  Pharmacies: Many pharmacies sell emergency contraception over the counter without requiring a prescription. You can simply walk in and ask for it at the pharmacy counter.  Health Clinics: Health clinics, including Planned Parenthood clinics and other community health centers, often provide emergency contraception. You may need to schedule an appointment or visit during specific hours.  Healthcare Providers: Your regular healthcare provider, such as a doctor or gynecologist, can prescribe emergency contraception for you. You may need to schedule an appointment to discuss your options and obtain a prescription.  Online Pharmacies: Some online pharmacies offer emergency contraception for purchase. Make sure to choose a reputable and licensed online pharmacy if you decide to go this route.  It's important to obtain emergency contraception as soon as possible after unprotected sex or contraceptive failure, as it is most effective when taken within the first 72 hours, although it may still be effective up to five days afterward, depending on the type of pill. If you have concerns or questions about emergency contraception, don't hesitate to reach out to a healthcare provider or pharmacist for guidance. |
| Can you find a location near me to get emergency contraception? | I'm sorry, but I don't have the capability to browse the internet or access specific locations. To find a location near you to get emergency contraception, you can try the following steps:  **Local Pharmacies:** Visit or call local pharmacies in your area. Many pharmacies carry emergency contraception over the counter.  **Healthcare Providers:** Contact your healthcare provider or gynecologist. They can provide guidance and may prescribe emergency contraception if needed.  **Family Planning Clinics:** Look for family planning clinics, sexual health clinics, or community health centers. They often offer emergency contraception services.  **Online Maps and Directories:** Use online maps or directories to search for nearby pharmacies or clinics that provide emergency contraception. You can use search engines or dedicated healthcare directories.  **Call a Health Hotline:** In some regions, there may be health hotlines that can provide information on where to obtain emergency contraception. Check with your local health department or family planning organizations.  Remember, time is a critical factor when it comes to emergency contraception. The sooner you take it after unprotected sex or contraceptive failure, the more effective it is likely to be. If you're unsure or have questions, don't hesitate to reach out to a healthcare professional for guidance. | As an AI language model, I don't have access to real-time location data or the ability to browse the internet. However, I can provide you with some general guidance on how to find a location near you to get emergency contraception:    1. **Search Online**: You can use search engines like Google or Bing to search for nearby pharmacies, health clinics, or Planned Parenthood locations. Use search terms like "emergency contraception near me" or "pharmacies near me" to find relevant options.    2. **Call Pharmacies or Clinics**: Once you have a list of nearby pharmacies or clinics, you can call them to inquire about their availability of emergency contraception and any specific requirements for obtaining it.    3. **Use Health Service Directories**: Some websites and apps offer directories of healthcare providers, including pharmacies and clinics that offer emergency contraception. You can search by location and filter the results to find nearby options.    4. **Ask for Recommendations**: If you have friends, family members, or acquaintances who have used emergency contraception before, you can ask them for recommendations on where to go.    5. **Check with Your Healthcare Provider**: If you have a regular healthcare provider, such as a doctor or gynecologist, you can contact their office and ask for guidance on where to access emergency contraception in your area.    Remember to act promptly, as emergency contraception is most effective when taken as soon as possible after unprotected sex or contraceptive failure. If you're unable to find a location nearby or have any difficulties, don't hesitate to seek assistance from a healthcare provider or pharmacist. | I'm unable to provide real-time location-based information or search for specific locations. To find a nearby location to get emergency contraception, consider using online maps, searching for pharmacies or family planning clinics in your area, or contacting local healthcare providers. If you have concerns or need immediate assistance, you may also call a local pharmacy or healthcare hotline for guidance. | I'm unable to access real-time location data or provide specific locations for emergency contraception. However, you can easily find nearby locations to obtain emergency contraception by using online resources such as:  Google Maps: You can search for pharmacies or health clinics in your area using Google Maps. Simply enter your location and search for "pharmacy" or "health clinic" to find nearby options.  Planned Parenthood: The Planned Parenthood website has a location finder tool where you can search for nearby Planned Parenthood health centers that offer emergency contraception and other reproductive health services.  Local Health Department Websites: Your local health department's website may provide information on where to access emergency contraception in your area.  Telehealth Services: Some telehealth services offer consultations and prescriptions for emergency contraception online. You can search for reputable telehealth providers in your region.  If you're unable to find a nearby location or have difficulty accessing emergency contraception, consider reaching out to a healthcare provider or pharmacist for assistance. They can provide guidance and help you find the nearest available options. |
| Is emergency contraception covered by insurance? how much does it cost? | Emergency contraception is generally available over the counter without a prescription, and its cost can vary depending on the brand and the place of purchase. Many insurance plans do cover emergency contraception, but coverage can vary, so it's important to check with your specific insurance provider.  Here are some points to consider:  **Over-the-Counter Availability:** Emergency contraception is available without a prescription at pharmacies, and you can purchase it directly from the shelf. It's often located in the family planning or women's health section.  **Insurance Coverage:** While many insurance plans cover emergency contraception, it's advisable to contact your insurance provider directly to confirm coverage and understand any potential out-of-pocket costs.  **Generic vs. Brand Name:** Generic versions of emergency contraception are often available and may be less expensive than brand-name options. Both generic and brand-name products are equally effective.  **Planned Parenthood and Health Clinics:** If you're concerned about the cost, consider checking with family planning clinics, health centers, or organizations like Planned Parenthood. They may offer emergency contraception at a reduced cost based on your income and circumstances.  **Coupons and Discounts:** Some pharmacies and manufacturers may offer coupons or discounts for emergency contraception. Check with the pharmacy or visit the manufacturer's website for any available savings programs.  Keep in mind that pricing and insurance coverage can vary based on your location and the specific policies of your insurance provider. If you have insurance, the best way to determine coverage and potential costs is to contact your insurance company directly. Additionally, healthcare providers or pharmacists can provide information about affordable options and potential assistance programs. | Emergency contraception is often covered by insurance, but coverage can vary depending on your insurance plan and location. Some insurance plans fully cover the cost of emergency contraception, while others may require a copayment or may only cover a portion of the cost.    If you have health insurance, you can contact your insurance provider or check your plan's details to see if emergency contraception is covered and what out-of-pocket costs you might incur. You can typically find this information on your insurance provider's website or by calling their customer service hotline.    If you don't have insurance or if your insurance doesn't cover emergency contraception, you can still purchase it over the counter at pharmacies or through healthcare providers. The cost of emergency contraception can vary depending on the brand and where you purchase it. On average, it may range from $30 to $50 for a single dose, although prices may vary.    Some pharmacies and clinics offer generic versions of emergency contraception, which may be less expensive than brand-name options. Additionally, Planned Parenthood and other clinics may offer emergency contraception at a reduced cost or on a sliding scale based on your income level.    If you're concerned about the cost of emergency contraception, you can also inquire about financial assistance programs or discounts that may be available through pharmacies or clinics in your area. | Emergency contraception is often covered by insurance, but coverage can vary based on your specific insurance plan. It's advisable to check with your insurance provider to understand the details of your coverage.  If you don't have insurance or if there are costs not covered, the price of emergency contraception can vary depending on the brand and where you obtain it. It's available over-the-counter in many places, and the cost typically ranges from $30 to $50. Some pharmacies may offer lower-cost generic versions. If cost is a concern, you can also inquire about lower-cost options or inquire about assistance programs at your local pharmacy or family planning clinic. | Emergency contraception is often covered by insurance plans, but coverage can vary depending on your specific insurance provider and plan. Some insurance plans fully cover the cost of emergency contraception, while others may require a copayment or coinsurance. Additionally, coverage may depend on factors such as your location and whether you have a prescription for the emergency contraception.  If you have insurance coverage and are considering obtaining emergency contraception, it's a good idea to contact your insurance provider directly to inquire about coverage details and any potential out-of-pocket costs.  If you don't have insurance or if emergency contraception isn't covered by your plan, you can expect to pay out-of-pocket for the medication. The cost of emergency contraception varies depending on the brand, type, and where you obtain it. Generally, it can range from $30 to $50 or more.  Some pharmacies and health clinics may offer reduced-cost or sliding-scale fees for emergency contraception based on your income level. Additionally, certain organizations and programs may provide free or low-cost emergency contraception to individuals in need.  If cost is a concern, consider contacting your local Planned Parenthood health center or community health clinic to inquire about affordable options for emergency contraception. They may be able to provide assistance or direct you to resources for obtaining the medication at a reduced cost. |
| What do I do if I missed my period after forgetting to take a day of my oral contraception? | Missing a period after forgetting to take a day of your oral contraception can be concerning, and there are a few steps you can take:  **Take a Pregnancy Test:**  If you've missed a period and there's a possibility of pregnancy due to a missed pill, take a pregnancy test. Home pregnancy tests are readily available at pharmacies and can provide you with quick results.  **Contact Your Healthcare Provider:**  If the pregnancy test is positive or if you have concerns about a missed period, contact your healthcare provider. They can provide guidance, confirm the pregnancy, and discuss your options.  **Resume Taking Pills as Prescribed:**  If the pregnancy test is negative and you're not pregnant, resume taking your birth control pills as prescribed. Use backup contraception (such as condoms) for the next 7 days to ensure continued protection.  **Discuss With Your Healthcare Provider:**  If you're consistently experiencing issues with remembering to take your pills or if you're concerned about the effectiveness of your current contraceptive method, discuss this with your healthcare provider. They can provide guidance on suitable options, including alternative forms of contraception.  **Consider Other Factors:**  Stress, illness, changes in weight, and other factors can also contribute to missed periods. Consider any additional factors that may affect your menstrual cycle.  It's important to remember that missing a period doesn't necessarily mean you are pregnant. However, if you have concerns, it's crucial to address them promptly. Contacting your healthcare provider and taking a pregnancy test are important steps in understanding your current situation and making informed decisions about your reproductive health. | If you missed a day of your oral contraception and subsequently missed your period, here are some steps you can take:    1. **Take a Pregnancy Test**: If you've missed your period after missing a dose of your oral contraception, the first step is to take a pregnancy test to determine whether you're pregnant. You can purchase a home pregnancy test from a pharmacy or visit a healthcare provider for testing.    2. **Continue Taking Birth Control**: Regardless of the results of the pregnancy test, continue taking your birth control pills as directed by your healthcare provider. Missing a dose of birth control can sometimes disrupt your menstrual cycle, so it's important to continue with your regular regimen to maintain contraceptive effectiveness.    3. **Contact Your Healthcare Provider**: If you're unsure about what to do or if you have concerns about your missed period, consider reaching out to your healthcare provider for guidance. They can provide personalized advice based on your individual situation and may recommend additional testing or evaluation if necessary.    4. **Consider Backup Contraception**: If you're concerned about the possibility of pregnancy, you may want to consider using backup contraception, such as condoms, until you've resolved any uncertainties about your missed period and contraceptive effectiveness.    5. **Monitor Symptoms**: Pay attention to any symptoms or changes in your body, such as nausea, breast tenderness, or fatigue, which could be early signs of pregnancy. If you experience any unusual symptoms or concerns, consult with your healthcare provider for further evaluation.    It's important to address missed doses of birth control promptly and take appropriate steps to minimize the risk of unintended pregnancy. If you're unsure about what to do or if you have any concerns, don't hesitate to seek guidance from a healthcare provider. | If you've missed a period after forgetting to take a day of your oral contraception, consider taking a pregnancy test to rule out pregnancy. Additionally, contact your healthcare provider to discuss your situation and seek guidance. They can provide personalized advice based on your health history and the specific details of your contraceptive use. It's essential to address any concerns about missed periods or potential pregnancy promptly with the assistance of a healthcare professional. | If you missed a day of your oral contraception and then missed your period, here are steps you can consider:  Take a Pregnancy Test: If you missed a day of your oral contraception and subsequently missed your period, it's important to take a pregnancy test to determine if you are pregnant. Pregnancy tests are available over the counter at pharmacies and are typically reliable when used correctly.  Consult Your Healthcare Provider: If you suspect you may be pregnant or if you have concerns about missing your period after missing a dose of your oral contraception, it's important to consult your healthcare provider. They can provide guidance, perform additional tests if necessary, and discuss your options moving forward.  Continue Taking Your Pills: Unless advised otherwise by your healthcare provider, continue taking your oral contraception pills as prescribed. Missing a period while taking oral contraception does not necessarily mean you are pregnant, as some factors, including stress, hormonal changes, and medication adjustments, can affect menstrual cycles.  Consider Backup Contraception: If you're concerned about the possibility of pregnancy after missing a dose of your oral contraception, consider using backup contraception, such as condoms, until you've discussed the situation with your healthcare provider and resolved any concerns.  Follow Up: Follow up with your healthcare provider as recommended to discuss the results of your pregnancy test, address any concerns or questions you may have, and determine the best course of action based on your individual circumstances.  Remember, missing a period after missing a dose of oral contraception can be concerning, but it doesn't necessarily indicate pregnancy. However, it's important to take appropriate steps to address any concerns and ensure your continued health and well-being. |
| What happens if I resume taking my pills but I really am pregnant? | If you resume taking your birth control pills but are already pregnant, continuing the pill is unlikely to harm the developing fetus. Birth control pills are generally considered safe during early pregnancy, and many women inadvertently continue taking them before realizing they are pregnant.  However, it's essential to consult with your healthcare provider if you suspect or confirm pregnancy, especially if you have been taking birth control pills. Your healthcare provider can provide guidance based on your specific situation, assess any potential risks, and help you make informed decisions.  Here are some important points to consider:  **Prenatal Care:** If you are pregnant, it's crucial to start prenatal care as soon as possible. Your healthcare provider can guide you on the necessary steps and screenings to ensure a healthy pregnancy.  **Stop Taking Birth Control Pills:** Once pregnancy is confirmed, your healthcare provider will likely advise you to stop taking birth control pills.  **Discuss Your Situation:** Share the details of your birth control use and any missed pills with your healthcare provider. This information is important for them to assess the situation and provide appropriate guidance.  **Alternative Birth Control Methods:** If you decide not to continue with the pregnancy or need to switch to a different form of contraception, discuss alternative birth control methods with your healthcare provider.  Remember that the information provided here is general, and individual circumstances can vary. It's crucial to seek guidance from a healthcare professional who can provide personalized advice based on your health history and specific situation. If you suspect pregnancy, contact your healthcare provider promptly to discuss your options and receive appropriate care. | If you resume taking your birth control pills after missing a dose but you are actually pregnant, it's important to understand that continuing to take birth control pills during pregnancy is generally not recommended and may not be effective in preventing or terminating the pregnancy. Here's what you should know:    1. **Birth Control Pills and Pregnancy**: Birth control pills are not intended to be used as a method of terminating or preventing pregnancy once you are already pregnant. While there is limited evidence to suggest that birth control pills taken during early pregnancy may not increase the risk of birth defects or miscarriage, they are not considered a safe or reliable method for terminating a pregnancy.    2. **Potential Risks**: Continuing to take birth control pills during pregnancy may expose the fetus to hormones that could potentially affect its development. Additionally, there is a risk of birth control pill failure, which could result in an unintended pregnancy continuing despite the use of birth control.    3. **Consult Your Healthcare Provider**: If you suspect that you may be pregnant after missing a dose of your birth control pills, it's important to consult with your healthcare provider as soon as possible. They can perform a pregnancy test and provide guidance on next steps based on your individual circumstances.    4. **Discuss Options**: If you are pregnant and do not wish to continue the pregnancy, your healthcare provider can discuss your options with you, which may include prenatal care, adoption, or abortion, depending on your preferences and the stage of pregnancy.    5. **Discontinue Birth Control**: If you are confirmed to be pregnant, your healthcare provider will likely advise you to discontinue taking birth control pills and may recommend alternative forms of prenatal care or contraception as needed.    Overall, if you suspect that you may be pregnant after missing a dose of birth control pills, it's important to seek prompt medical advice from your healthcare provider to determine the best course of action for your health and well-being. | If you resume taking your oral contraception but are actually pregnant, continuing to take the pills won't affect the pregnancy. However, it's crucial to inform your healthcare provider about the situation. They can guide you on the next steps, including potential adjustments to your healthcare plan. Early detection and consultation with a healthcare professional are important for managing a pregnancy or addressing any concerns related to contraceptive use. | If you resume taking your oral contraception pills but you are actually pregnant, it's important to consult with your healthcare provider as soon as possible. Here's what you should consider:  Stop Taking the Pills: If you discover that you are pregnant, you should stop taking your oral contraception pills immediately.  Consult Your Healthcare Provider: Contact your healthcare provider to schedule an appointment to confirm the pregnancy and discuss your options. They can provide guidance on prenatal care, discuss your options regarding the pregnancy, and address any concerns you may have.  Continue Prenatal Care: If you decide to continue the pregnancy, your healthcare provider will guide you through prenatal care and monitor your health and the health of the fetus throughout the pregnancy.  Considerations for Birth Control Pills: It's important to note that taking birth control pills while pregnant does not typically cause harm to the fetus. However, it's generally recommended to stop taking them once pregnancy is confirmed.  Discuss Potential Risks: Your healthcare provider can discuss any potential risks or concerns associated with taking birth control pills during early pregnancy and provide personalized recommendations based on your individual situation.  Address Emotional and Psychological Support: Discovering an unexpected pregnancy can be emotionally challenging. Consider seeking support from friends, family, or a counselor to help you navigate this situation.  Remember, if you suspect you may be pregnant while taking oral contraception pills, it's crucial to seek medical advice promptly to ensure you receive appropriate care and support. |
| Can you help me find a healthcare provider? | I'm sorry, but I don't have the capability to browse the internet or access specific databases, so I can't help you find a healthcare provider directly. However, I can provide guidance on how you might go about finding one:  **Primary Care Physician:** If you have a primary care physician, they can often provide guidance on reproductive health, family planning, and contraception. Contact your primary care doctor's office for assistance.  **Health Insurance Provider:** Check with your health insurance provider for a list of in-network healthcare providers. Insurance websites or customer service representatives can help you find healthcare professionals in your network.  **Online Directories:** Use online directories to search for healthcare providers in your area. Websites like Healthgrades, Zocdoc, or the American Medical Association's Doctor Finder allow you to search for doctors by specialty and location.  **Local Health Clinics:** Family planning clinics, community health centers, or women's health clinics may offer reproductive health services. Planned Parenthood is a well-known organization that provides a range of reproductive health services.  **Referrals:** Ask friends, family, or colleagues for recommendations. Personal referrals can be valuable in finding a healthcare provider who meets your needs.  **Online Reviews:** Read online reviews of healthcare providers in your area. While reviews should be taken with a grain of caution, they can provide insights into the experiences of other patients.  Once you have a list of potential healthcare providers, consider factors such as location, office hours, and whether they accept your insurance. Don't hesitate to schedule an appointment to meet with a healthcare provider and discuss your specific needs and concerns. | Certainly! Finding a healthcare provider can depend on various factors such as your location, insurance coverage, and specific healthcare needs. Here are some general steps to help you find a healthcare provider:    1. **Check with Your Insurance**: If you have health insurance, start by checking your insurance provider's website or contacting their customer service hotline to find a list of in-network healthcare providers. This can help you identify healthcare professionals who will be covered by your insurance plan.    2. **Use Online Directories**: There are many online directories that can help you find healthcare providers in your area. Websites like Healthgrades, Zocdoc, and WebMD's physician directory allow you to search for providers by location, specialty, and other criteria. You can often read reviews and see ratings from other patients to help you make an informed decision.    3. **Ask for Recommendations**: Ask friends, family members, or coworkers for recommendations. They may be able to suggest healthcare providers they trust and have had positive experiences with.    4. **Check with Local Hospitals or Clinics**: Hospitals and community health clinics often have directories of affiliated healthcare providers, including physicians, nurse practitioners, and other specialists. You can contact them directly or visit their websites to find more information.    5. **Consider Your Specific Needs**: Depending on your healthcare needs, you may want to find a primary care physician, gynecologist, specialist, or other healthcare professional. Consider what type of care you need and look for providers who specialize in that area.    6. **Research Credentials and Experience**: Once you've identified potential healthcare providers, take some time to research their credentials, experience, and any specialties or areas of expertise. You can often find this information on their websites or through professional licensing boards.    7. **Schedule an Appointment**: Once you've found a healthcare provider who meets your needs, contact their office to schedule an appointment. Be prepared to provide your insurance information and any relevant medical history.    By following these steps, you should be able to find a healthcare provider who can meet your needs and provide you with the care and support you require. If you have any specific preferences or requirements, don't hesitate to communicate them to the provider's office when scheduling your appointment. | I can't assist in finding specific healthcare providers. To find a healthcare provider, you may consider asking for recommendations from friends or family, using online directories, checking with your insurance provider for in-network options, or contacting local clinics or hospitals. Additionally, many regions have online platforms or directories that can help you find healthcare professionals based on your location and needs. | I can't browse the internet or access real-time databases to find specific healthcare providers. However, I can suggest some general steps to help you find a healthcare provider:  Use Online Directories: Websites like Zocdoc, Healthgrades, or WebMD's physician directory allow you to search for healthcare providers in your area based on criteria such as location, specialty, and insurance accepted.  Check with Your Insurance Company: If you have health insurance, your insurance provider's website may have a tool or directory to help you find healthcare providers who are in-network with your plan.  Ask for Recommendations: Reach out to friends, family members, or colleagues for recommendations. They may be able to recommend healthcare providers they have had positive experiences with.  Visit Community Health Centers: Community health centers often provide a range of healthcare services, including primary care, at reduced costs. You can search online or contact your local health department for information on community health centers in your area.  Consider Telemedicine: Telemedicine services allow you to consult with healthcare providers remotely. Many telemedicine platforms offer virtual appointments with licensed healthcare professionals.  Contact Local Hospitals: Hospitals often have affiliated clinics or outpatient centers where you can receive primary care or specialized services. You can contact the hospital's main line or visit their website for more information.  Once you've identified potential healthcare providers, consider factors such as their location, specialties, availability, and patient reviews to help you make an informed decision. Don't hesitate to schedule a consultation or appointment to meet with the provider and determine if they are a good fit for your healthcare needs. |
| What kind of healthcare provider do I need to see for this issue? | For issues related to contraception, missed periods, and reproductive health, you may want to see a healthcare provider with expertise in women's health and reproductive medicine. Here are some types of healthcare providers you can consider:  **Obstetrician-Gynecologist (OB-GYN):**  An OB-GYN is a medical doctor who specializes in women's reproductive health. They can provide a wide range of services, including contraception counseling, prenatal care, and gynecological exams.  **Family Medicine Physician:**  Family medicine physicians are primary care doctors who provide comprehensive healthcare to individuals of all ages, including women's health. They can address a variety of reproductive health concerns and provide contraception counseling.  **Women's Health Nurse Practitioner:**  Nurse practitioners specializing in women's health can provide a range of reproductive health services, including contraception counseling, gynecological exams, and family planning.  **Reproductive Endocrinologist:**  If you are experiencing difficulties with fertility or have concerns about reproductive health beyond contraception, a reproductive endocrinologist is a specialist in the field of fertility and reproductive medicine.  **Planned Parenthood or Family Planning Clinic:**  Planned Parenthood and other family planning clinics often provide affordable reproductive health services, including contraception counseling, screenings, and family planning.  When choosing a healthcare provider, consider factors such as their expertise, the services they offer, and whether they accept your insurance. It's important to have open communication with your healthcare provider about your concerns, questions, and any specific issues you may be experiencing.  If you're unsure about which type of provider is most appropriate for your situation, you can start by scheduling an appointment with a primary care physician who can provide guidance and, if necessary, refer you to a specialist. | For issues related to birth control, missed periods, or concerns about pregnancy after missing doses of oral contraception, you may want to consider seeing one of the following types of healthcare providers:    1. **Primary Care Physician (PCP)**: Your primary care physician can address a wide range of general health concerns, including issues related to contraception and reproductive health. They can assess your overall health, review your medical history, and provide guidance on managing missed doses of oral contraception or concerns about pregnancy.    2. **Gynecologist**: A gynecologist specializes in women's reproductive health, including contraception, menstrual issues, and pregnancy. They can provide comprehensive care related to birth control, perform pelvic exams, order tests such as pregnancy tests or blood work, and offer guidance on managing missed doses of oral contraception.    3. **Women's Health Nurse Practitioner (WHNP)**: WHNPs are advanced practice nurses who specialize in women's health. They are trained to provide a wide range of services, including contraceptive counseling, reproductive health screenings, and management of menstrual irregularities.    4. **Family Planning Clinic**: Family planning clinics, such as those operated by Planned Parenthood or other organizations, specialize in providing reproductive health services, including contraception, pregnancy testing, and counseling. They often offer affordable or sliding-scale fees for services.    5. **Obstetrician-Gynecologist (OB-GYN)**: OB-GYNs are medical doctors who specialize in both obstetrics (pregnancy and childbirth) and gynecology (women's reproductive health). They can address a variety of concerns related to contraception, menstrual issues, and pregnancy.    When choosing a healthcare provider for this issue, consider your personal preferences, such as whether you prefer a male or female provider, the provider's location and availability, and any specific concerns or questions you have. Additionally, if you have a primary care physician or established relationship with a healthcare provider, you may feel comfortable discussing these issues with them. | For issues related to reproductive health, missed periods, or concerns about contraception, you may want to see a healthcare provider specializing in gynecology or obstetrics. A gynecologist is a doctor who specializes in women's reproductive health, while an obstetrician focuses on pregnancy and childbirth. You can also start by scheduling an appointment with your primary care physician, who can assess your situation and refer you to a specialist if needed. | To determine the type of healthcare provider you should see for your specific issue, it depends on the nature of the issue. Here are some common scenarios and the corresponding healthcare providers you might consider:  Primary Care Physician (PCP): If you have a general health concern, need routine check-ups, or want to discuss preventive care, your primary care physician is typically the first point of contact. They can address a wide range of health issues and provide referrals to specialists if needed.  Obstetrician/Gynecologist (OB/GYN): For reproductive health concerns such as birth control, pregnancy, menstrual irregularities, or gynecological conditions, an OB/GYN specializes in women's health and can provide comprehensive care and guidance.  Endocrinologist: If your issue is related to hormonal imbalances, such as thyroid disorders, diabetes, or polycystic ovary syndrome (PCOS), an endocrinologist specializes in diagnosing and treating hormonal conditions.  Urologist: For issues related to the urinary system or reproductive organs in men, such as erectile dysfunction, infertility, or prostate problems, a urologist specializes in these areas and can provide appropriate evaluation and treatment.  Dermatologist: If your issue involves skin, hair, or nail conditions, such as acne, eczema, or hair loss, a dermatologist specializes in diagnosing and treating dermatological issues.  Psychiatrist or Psychologist: If your issue is related to mental health, such as depression, anxiety, or stress management, a psychiatrist or psychologist specializes in diagnosing and treating mental health conditions and can provide therapy or medication management.  Specialist: Depending on the specific nature of your issue, you may need to see a specialist in a particular field. For example, if you have chronic pain, you might see a pain management specialist; if you have digestive issues, you might see a gastroenterologist.  Ultimately, the type of healthcare provider you should see depends on the nature of your issue and your individual healthcare needs. If you're unsure, you can start by scheduling an appointment with your primary care physician, who can assess your situation and provide appropriate referrals or guidance. |
| *Asked ten minutes to one week later*  Do you remember our conversation about oral contraception? | Yes, I have access to the conversation history, and I remember our discussion about oral contraception. If you have any more questions or if there's anything specific you'd like to know or discuss further regarding oral contraception or any other topic, feel free to let me know! | I can't recall past interactions, but I'm here to help you with any questions or discussions you have about oral contraception. What would you like to know or discuss. | I don't have the capability to remember past interactions or conversations due to privacy and security reasons. Each session is stateless and doesn't retain information from previous conversations. If you have questions or need information, feel free to ask, and I'll do my best to assist you. | Yes, I can remember our conversation about oral contraception. We discussed various aspects, including what to do if a dose is missed, the importance of consulting a healthcare provider, and considerations if you suspect you might be pregnant. If you have any more questions or need further assistance on this topic, feel free to ask! |

**Table S3.** Additional ChatGPT inquiry.

| **Account 1 Question** | **ChatGPT Response** |
| --- | --- |
| Can you tell how proficient in English I am? | I don't have the ability to assess your proficiency in English or any other language. My responses are generated based on patterns and information present in the data on which I was trained. If you have any specific language preferences or if there's a particular way you'd like me to communicate, feel free to let me know, and I'll do my best to accommodate your preferences. |
| Can you tell me about birth control at a fourth-grade English proficiency level? | Absolutely! Birth control is a way for grown-ups to plan when they want to have a baby. It helps them make decisions about how many children they want and when it's the right time. There are different types of birth control, like pills, patches, and devices that help prevent pregnancy. These methods work by stopping the woman's egg from meeting a man's sperm.  Some people also use condoms, which are like special covers for the private parts, to help prevent pregnancy and protect against diseases. It's essential for grown-ups to talk to a doctor about which type of birth control is best for them. Remember, it's a grown-up topic, and kids may not need to worry about it until they are much older. If you have questions, it's a good idea to talk to your parents or a trusted adult who can give you the right information when the time is right. |
| What is your default English proficiency level for your responses to me? | I aim to provide responses at a level suitable for a high school English proficiency. However, I can adjust my language complexity based on your preferences. If you'd like simpler explanations or more advanced language, feel free to let me know, and I'll adapt accordingly! |
| What should I do if I missed a day of my oral contraception birth control? Answer at fourth grade English proficiency level. | If you missed taking your birth control pill, you should tell an adult or go to a doctor. They can help you figure out what to do next. It's important to take your pills every day to stay safe and prevent pregnancy. |
| Can you show me visuals instead of word answers? | I'm sorry, but I can't display visuals as I'm a text-based AI model. I can, however, describe or explain things in more detail through text. If you have a specific topic or concept you'd like me to explain, I'll do my best to provide a clear and informative description using words. |
| When was you last training cut off date? | My training data includes information up until January 2022. Any events, developments, or data beyond that date are not part of my training and knowledge base. |
| Can you cite the source of where you receive your information from? | I don't have direct access to my training data or know where it came from. I was trained on a diverse range of sources from the internet, such as articles, books, and websites, to develop a broad understanding of human language. I do not have the ability to provide specific citations for individual pieces of information or attribute them to particular sources. If you have specific questions about a topic, feel free to ask, and I'll do my best to provide information based on my training. Keep in mind that while I strive to provide accurate and up-to-date information, my responses may not always reflect the most current news events or developments. |


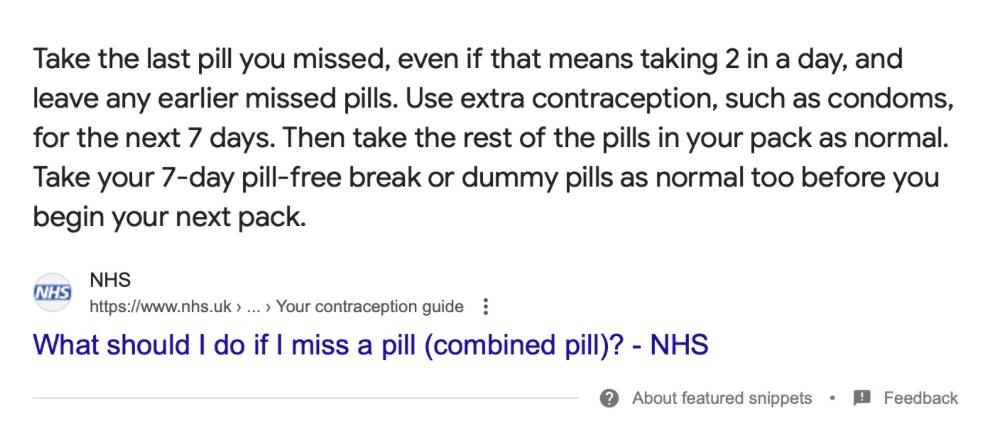


**Figure S1.** The first snippet result upon entering “what should I do if I missed a day of my oral contraception birth control?” in Google Search.


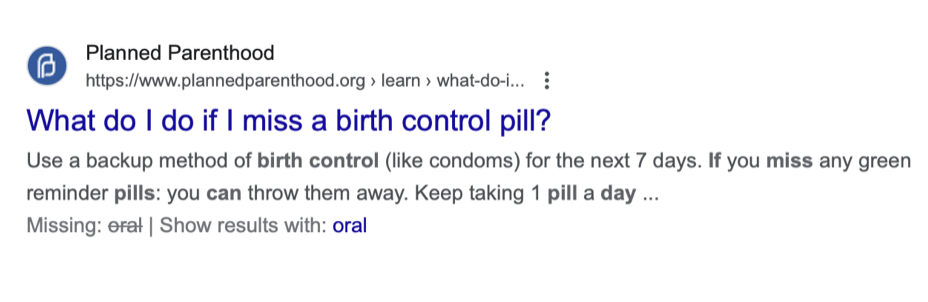


**Figure S2.** The second snippet result upon entering “what should I do if I missed a day of my oral contraception birth control?” in Google Search.


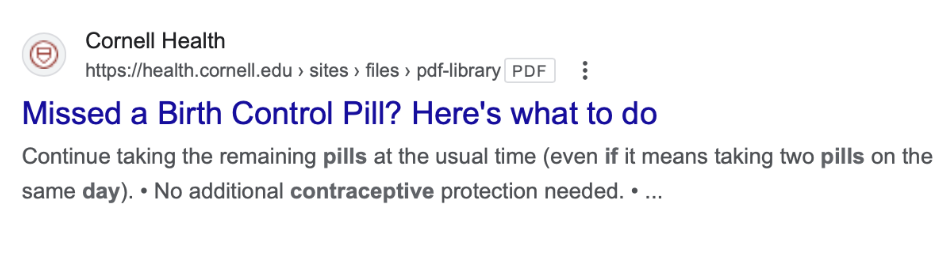


**Figure S3.** The third snippet result upon entering “what should I do if I missed a day of my oral contraception birth control?” in Google Search.


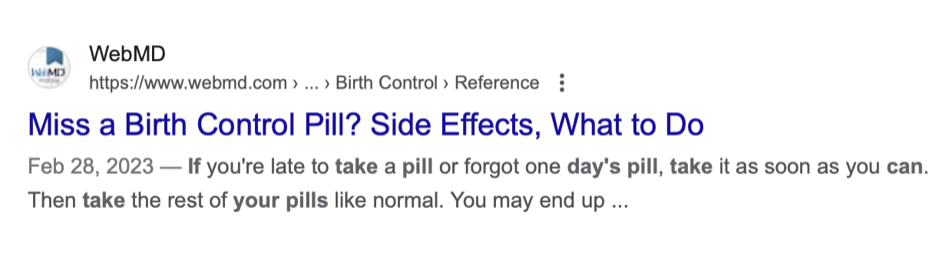


**Figure S4.** The fourth snippet result upon entering “what should I do if I missed a day of my oral contraception birth control?” in Google Search.


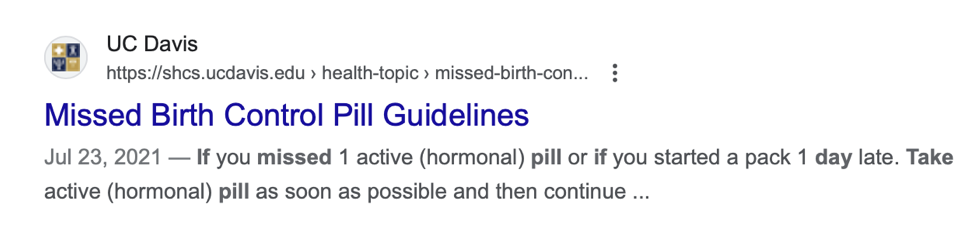


**Figure S5.** The fifth snippet result upon entering “what should I do if I missed a day of my oral contraception birth control?” in Google Search.


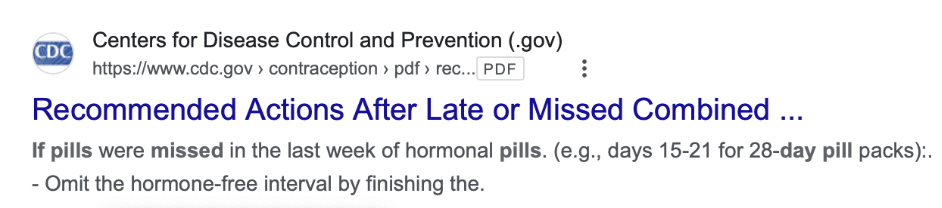


**Figure S6.** The sixth snippet result upon entering “what should I do if I missed a day of my oral contraception birth control?” in Google Search.


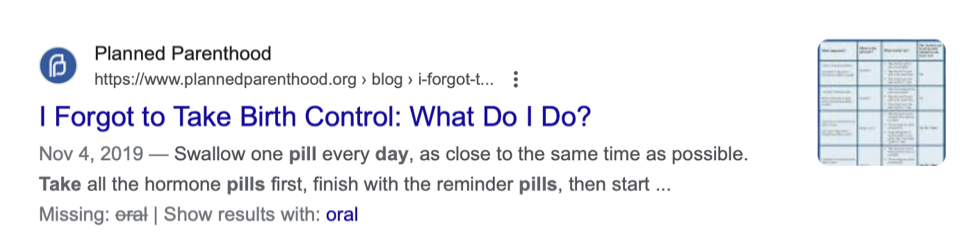


**Figure S7.** The seventh snippet result upon entering “what should I do if I missed a day of my oral contraception birth control?” in Google Search.
